# Kidney Modelling for FDG Excretion with PET

**DOI:** 10.1155/2007/63234

**Published:** 2007-07-02

**Authors:** Huiting Qiao, Jing Bai, Yingmao Chen, Jiahe Tian

**Affiliations:** ^1^Department of Biomedical Engineering, Tsinghua University, Beijing 100084, China; ^2^Department of Nuclear Medicine, General Hospital of PLA, Beijing 100853, China

## Abstract

The purpose of this study was to detect the physiological process of FDG's filtration from blood to urine and to establish a mathematical model to describe the process. Dynamic positron emission tomography scan for FDG was performed on seven normal volunteers. The filtration process in kidney can be seen in the sequential images of each study. Variational distribution of FDG in kidney can be detected in dynamic data. According to the structure and function, kidney is divided into parenchyma and pelvis. A unidirectional three-compartment model is proposed to describe the renal function in FDG excretion. The time-activity curves that were picked up from the parenchyma, pelvis, and abdominal aorta were used to estimate the parameter of the model. The output of the model has fitted well with the original curve from dynamic data.

## 1. INTRODUCTION

The development of positron emission tomography (PET) has made it possible to detect the physiological process in a human body. [18F]fluoro-2-deoxy-D-glucose(FDG) is the analog of glucose, which is widely used in clinical PET experiment [1]. In order to understand the metabolism of glucose and to detect diseases better, mathematical models of FDG have been established for brain, heart, liver, and some other organs [2–5]. Although kidney is the most important organ in the metabolism system of a human, and large quantity of FDG in the body is accumulated in the urine through the kidney [6], yet little work has been done for kidney modelling with FDG PET. There are two major reasons why only a few mathematical models are established for kidney. The first reason is because of the complicated structure and function of the kidney [7], and the second reason is due to the high excretion of FDG through the kidney [6, 8]. FDG, unlike glucose, cannot be reabsorbed in the proximal tubules of the kidney, and so FDG will be accumulated in the urine.

To describe the filtration process of FDG from blood to urine, seven normal volunteers took part in the dynamic FDG-PET experiment. The imaging data has been used for kinetic analysis and parameter estimation.

Compared to the high concentration of FDG in kidney collection system, the small quantity of metabolized FDG in kidney can be neglected. The dynamic imaging shows the filtration of FDG and the process of urinary excretion. Though three-compartment four-rate model is widely used to describe the metabolism of some of the human organs, it is not suitable for describing kidney. A unidirectional compartment model is proposed to show the transport process of FDG from blood to urine. Due to the kidney which contains great quantity of blood vessel and collection system of urine, the effect fractions from the blood and the urine to parenchyma will be all considered in the model.

Bouchet et al. [7] had proposed the model which divides the kidney into five parts in order to compute the absorbed fractions of radiopharmaceuticals. In our study of dynamic PET imaging, the inhomogeneity of kidney can also be seen. Here, the kidney is separated into two parts: parenchyma and pelvis. Time-activity curves are picked up from each part and are used to estimate the parameters. Though there are great differences between each set of parameters, the output of the model is basically in accord with the original curve.

## 2. MATERIALS AND METHOD

### 2.1. Subjects

Seven normal volunteers participated in the study. The age of volunteers is between 34 to 60 years (mean ± SD, 47 ± 11 years), the height is from 165 to 185 cm (172 ± 7 cm), and the weight is from 53 to 94 kg (76 ± 12 kg). None of them has had a prior history of any major metabolic illnesses or renal diseases. Dynamic FDG-PET scans were performed on each volunteer. They were asked to fast for at least four hours and to empty the bladder before the scanning. During the experiment, they were asked to lie down still and to keep quiet. Each volunteer was informed fully about the purposes and procedures of the study and was asked to give a written consent.

### 2.2. PET scanning protocol

All the experiments were done with an ECAT EXACT HR^+^ PET (CTI/Siemens, Inc., TN, USA). The scanner provides 63 continuous transaxial slices with a 15.5 cm field of view. The spatial resolution is 4.2 mm full width at half maximum in the centre field of view. The experiments were performed in a single bed position covering the kidneys. The tracer dose of FDG, 4-5 mCi, was injected intravenously into the human body, and PET scan began immediately after the injection. In the sampling protocol, the dynamic imaging sequences consisted of six 10-seconds frames; eight 20-seconds frames; six 30-seconds frames; five 60-seconds frames; four 300-seconds frames; and three 600-seconds frames, which is in total of 32 frames for a total scan time of 61 minutes and 40 seconds.

### 2.3. Organ time-activity measurement

Kidney and abdominal aorta can be detected from the dynamic PET image. During study, the time-activity curve was picked up from the drawn region of interest (ROI) in each frame. The radioactivity was calculated by averaging the whole voxel's values within the ROI. Kidney is different from other organs, because it is heterogeneous for the complex physiological function. Kidney is divided into two parts: renal parenchyma and pelvis. In this study, the ROIs of parenchyma and pelvis were drawn for each plan from the 3D image data in separate frames which have a best view of certain parts of the kidney. The ROI of parenchyma is drawn in the frame for about 2 minutes after the scan, and the ROI of pelvis is drawn in the frame for about 5 minutes. The blood time-activity curve (BTAC) was derived from the ROI in abdominal aorta [9], which is drawn in the frame for 1 minute.

### 2.4. Model analysis

**Figure 1:**
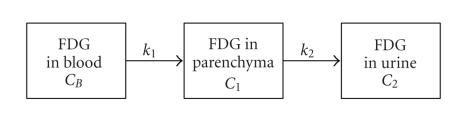
Model of kidney for FDG excretion.

A three-compartment model (Figure 1) with four parameters is proposed to simply describe the excretion of FDG. In this simple model, blood, renal parenchyma, and urine compartments are assumed to be uniformly distributed with FDG, respectively. The urine compartment includes urine in the pelvis and urine in the bladder. For the high excretion of FDG, the metabolism of FDG in kidney is unobvious and is neglected in the model. *k*_1_ and *k*_2_ are the rate constants of FDG between each compartment,

(1) $$\frac{dC_{1}}{dt}=k_{1}C_{B}-k_{2}C_{1},$$

(2) $$C_{1}\left(t\right)=k_{1}\cdot e^{-k_{2^{t}}}⊗C_{B}\left(t\right),$$

(3) $$C_{T}\left(t\right)=k_{1}e^{-k_{2}t}⊗C_{B}\left(t\right)+f_{1}C_{B}\left(t\right)+f_{2}C_{\text{pelvis}},$$

where *C*_*B*_ is the concentration of FDG in blood, *C*_1_ is the concentration of FDG in parenchyma, *C*_2_ is the concentration of FDG in urine, *C*_pelvis_ is the concentration of FDG in pelvis, and *C*_*T*_ is the concentration of FDG detected from PET. Equation (1) shows the kinetic description of the compartment model, (2) is derived from (1), and (3) shows that the activity in kidney detected by PET is not only decided by *C*_1_, but also affected by *C*_*B*_ and *C*_pelvis_. Kidney is an organ which is rich in blood. So, the parameter *f*_1_ is used to describe the effect fraction from the blood to parenchyma. Parenchyma and pelvis are so close to each other inside the kidney that their effect on each other cannot be neglected. Thus, parameter *f*_2_ is introduced to calibrate the effect of the urine from the pelvis.

## 3. RESULT

### 3.1. FDG imaging

**Figure 2:**
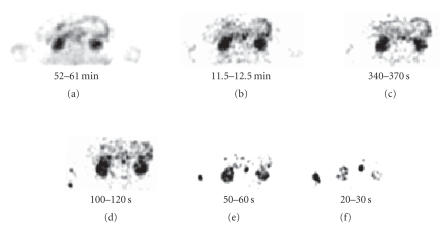
Some of the sequential transaxial images of one study.

**Figure 3:**
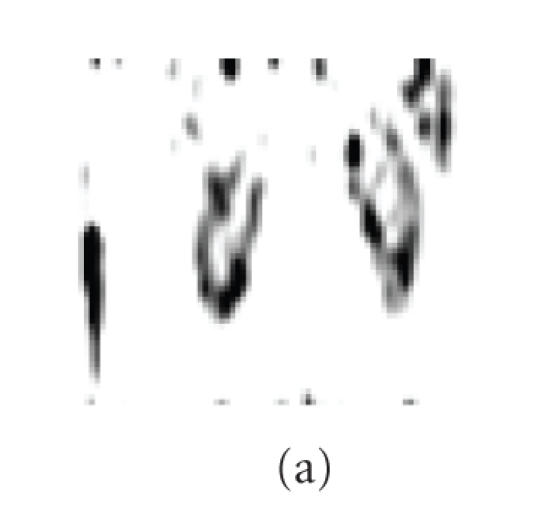

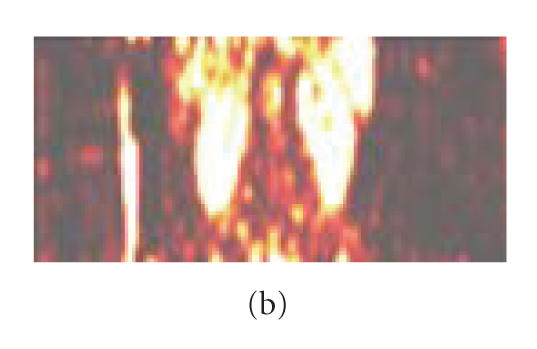

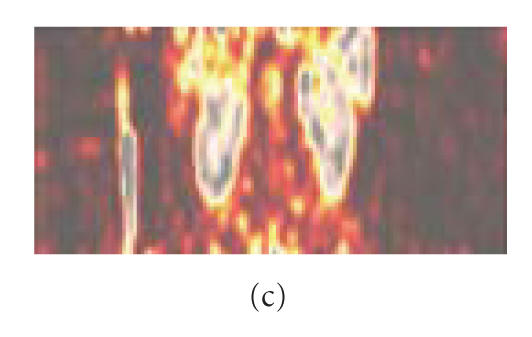

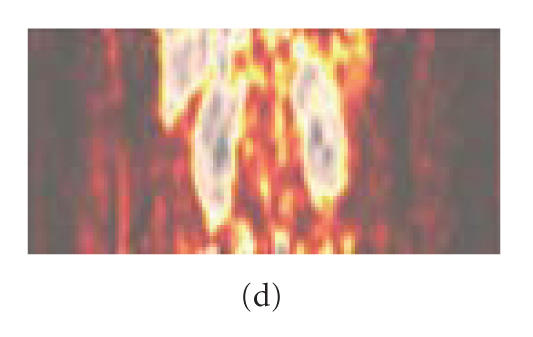
Images of kidney: (a)-(b) is one coronal section at 40–50 seconds, (c) is the fused image of (a) and (b), and (d) is another fused coronal section at 310–340 seconds.

In these seven subjects, the kidneys are clearly visualized with very high target-to-background ratio (Figure 2). Shreve et al. [10] had used carbon-11-acetate as the tracer to detect kidney. In their studies, no urinary tracer activity has appeared in the intrarenal collecting system. Unlike carbon-11-acetate and glucose, FDG is a kind of tracer which cannot be reabsorbed when the initial urine passes through the renal tubule. Thus, FDG can be detected in renal pelvis in some frames. The concentration distribution variation can be seen in kidney in different frames. Figures 3(a) and 3(b) are the same coronal sections of a dynamic PET study in one frame (in 1 minute after injection), but the two images are in different brightness (window center) and contrast (window wide). The part of the kidney in which activity is highly accumulated can be found by adjusting brightness and contrast (Figure 3(a)). Figure 2(b) gives the outline of the whole organ in hot color scheme, and two images, Figures 3(a) and 3(b) were fused. It can be seen from the fused image (Figure 3(c)) that in early time after the injection, the FDG is mostly accumulated in the edge of the kidney, where the renal cortex and some of renal medulla are located. Figure 3(d) is another fused image in frame for over 5 minutes after injection. It can be seen that the high activity concentration appears in the renal depression, where the position of renal pelvis is.

### 3.2. Kinetic parameter

Seven dynamic data sets from the seven subjects were used for parameter estimation. The BTAC which has been picked up from aorta and the tissue time-activity curve (TTAC) of pelvis is the input of the model, while the detected TTAC of the parenchyma is the output of the model. Weighted least squares principle [11] was used to fit the simple kidney model. The weight is the inverses of the measurement error. Parameters for the model are listed in Table 1.

**Figure 4:**
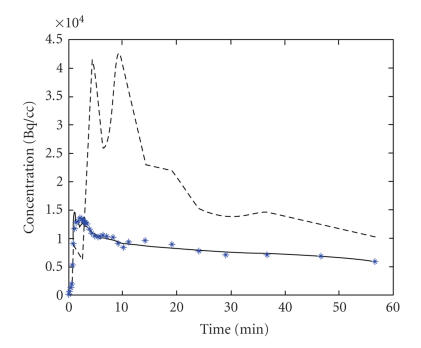
Time-activity curve. The normal line represents the time-activity curve of renal parenchyma, the dashed line is the time-activity curve of renal pelvis, and the asterisks show the point we fitted.

The average and standard deviations (SD) for *k*_1_, *k*_2_, *f*_1_, and *f*_2_ are also shown in Table 1. The average rate constant *k*_1_ is 1.5822 min^−1^, and *k*_2_ is 1.5795 min^−1^. The effect fraction *f*_1_ is 0.1269, and *f*_2_ is 0.0491. The parameters of each subject are compared with each other. Results show significant differences in the parameters for the subjects. Characters such as age, height, or weight of the individual subjects may be one of the reasons for the differences in the parameters. The confirmations of ROI for each study also lead to the huge differences especially for *f*_2_, which describes the effect from urine of pelvis to renal parenchyma. The further the ROI of the parenchyma is from the pelvis, the smaller *f*_2_ will be. Figure 4 shows the time-activity curve of one of the experiments. The asterisk points are the result from compartment model and estimated parameters.

## 4. DISCUSSION

The research of metabolism with FDG PET has been done for many years, but only a little work is focused on kidney. In some study, the function of kidney is just described by a constant rate from plasma to urine [5]. For some tracer, kidney can also be described by the classical three-compartment model [12]. The kidney model for FDG is different, because F-18 FDG is excreted greatly into the tubular lumen and accumulated in the renal collecting system [8], no reabsorption appears. So we use only one-direction compartment model to describe kidney. Seven sets of dynamic clinical data were being used to estimate the parameters. Results have shown great differences in each subject. However, the output of the model fits well with the original curve from clinical data.

In order to make the model simple and workable, some assumptions were made in this study: the blood time-activity curve which was picked up from the aorta is used as plasma time-activity curve in parameter estimation; no urine is accumulated in the parenchyma, and the effect from the pelvis is assumed to be consistent.

The high excretion of FDG has made it difficult to analyze the glucose metabolism of kidney and also to detect renal diseases. However, the high excretion of FDG can still provide other important physiological information. In the dynamic data, Parenchyma and pelvis can be distinguished, and the time-activity curves are shown in Figure 4. The peaks of the two curves which show the highest concentration appear at different time points. The peak of the pelvis appears a little later than that of parenchyma. The result is in accord with the renal physiology. Two peaks are shown in the time-activity curve for pelvis. This can be explained by the physiology of pelvis. Pelvis is the tissue which accumulates urine temporarily. The urine is then transported to bladder. The time-activity curve of the pelvis shows the process of urinary transport to bladder. The process cannot just be described by a rate constant. Hence, this model is just a preliminary study of kidney, further investigation will be done.

## Figures and Tables

**Table 1:** Parameters of the kidney model.

	*k*_1_(min^−1^)	*k*_2_(min^−1^)	*f*_1_	*f*_2_

Subject1	3.4659	2.8042	0.15964	0.07574
Subject2	1.8423	2.3827	0.03293	0.10977
Subject3	1.3318	1.9806	0.19699	0.04073
Subject4	0.7703	0.8280	0.17543	0.03623
Subject5	1.5503	1.1120	0.10181	0.00000
Subject6	0.8981	0.9486	0.07342	0.04563
Subject7	1.2170	1.0007	0.03525	0.03525
Average	1.5822	1.5795	0.1269	0.0491
SD	0.9074	0.7981	0.0593	0.0347
